# Interplay of pandemic and seasonal parameters in dental emergency service

**DOI:** 10.1186/s12903-022-02524-4

**Published:** 2022-11-08

**Authors:** Charlott Luise Hell, James Deschner, Pablo Cores Ziskoven, Philipp Mildenberger, Jens Weusmann

**Affiliations:** 1 Department of Periodontology and Operative Dentistry, University Medical Center of the Johannes Gutenberg University, Augustusplatz 2, 55131 Mainz, Germany; 2 Institute of Medical Biostatistics, Epidemiology and Informatics (IMBEI), University Medical Center of the Johannes Gutenberg University, Mainz, Germany

**Keywords:** COVID-19, Dental emergencies, Emergency patients, Seasonal effects, COVID-19 waves

## Abstract

**Background:**

To evaluate the relationship between pandemic events and dental emergency service frequentation. Utilization patterns in the scope of the COVID-19 pandemic were analyzed and considered in regard of seasonal parameters.

**Methods:**

All outpatients seeking treatment in a university hospital’s dental emergency service were analyzed in the years 2019, 2019 and 2020 according to demographic data and emergencies were subdivided into “absolute” and “relative”. The years 2018 and 2019 were used to compare COVID-19 and non-COVID-19 phases. Defined waves of the pandemic were compared with equivalent prior-year periods.

**Results:**

Our study includes 11,219 dental emergency patients over a period of three years with a slight surplus of male patients. Comparing the pre-COVID-19 years and 2020 as a year of pandemic, the total count of cases decreased by more than 25%. The share of absolute emergencies in 2020 was higher than in the years before (*p* < 0.0001). The under-utilization during the waves was more pronounced during the first wave compared to the second waves.

**Conclusions:**

Additionally to the clear decrease by more than 25% in 2020, we found an inverse dependency of 7-day-incidence of COVID-19 and number of visits. This effect was more distinct for relative emergencies, while the number of patients with absolute emergencies remains rather constant. Probably, there is an acclimatization effect regarding the waves. Patients older than 60 years who suffered from relative emergencies showed an under-utilzation during 2020. During a pandemic such as COVID-19, the effect of under-utilization is more pronounced among elderly patients. However, a pandemic acclimatization effect seems to occur. This can be taken into account in the administration of this kind of circumstances in the future.

## Background

Until January 2^nd^, 2021, there have been more than 1,755,351 confirmed cases of COVID-19 and more than 33,960 associated deaths in Germany [1]; until April 1^st^ 2022 21,357,039 confirmed cases were registered [2].

The public COVID-19 infection prevention led to different governmentally regulated measures to slow down the rate of infection. Those regulations included several lockdown measures in public life; pandemic-related restrictions had an impact on the daily lives of all citizens in Germany. All healthcare facilities were distinctly hampered by the COVID-19 pandemic around the world; additionally, dentistry as a potential aerosol-forming sector was of particular interest [3]. The specific and unspecific governmental measures may very likely have resulted in a COVID-19 pandemic quantitative and qualitative impact on dental health behavior such as emergency visits.

The dental emergency service is regulated by the dental boards in Germany and is conducted by private practices and university hospitals. The relative indications for emergency treatment do not represent an emergency in an actual sense and, objectively, no urgent treatment is required [4]. Nonetheless, since patients are usually not able to distinguish, out of hours treatment can be indicated even in relative emergencies.

The absolute emergencies correspond to the indications, which imply an urgent treatment.

The aim of our study was to investigate the hitherto barely known influence of a pandemic situation and, specifically, its wave-formed process on visiting behavior and emergency characteristics in dental emergency service. We intended to analyze the changes in patient behavior focusing on the first two waves on virus outbreak taking into account annually rhythmic events of the year such as vacations and seasons.. The insights gained from this study may provide information about the demands for healthcare services in future similar situations. Furthermore, this study can help to assess the general challenges of the dental emergency outpatient service.

## Methods

### Emergency service

This study included the entirety of outpatients who received treatment beyond the regular consultation hours in the dental emergency service of the University Mainz, Germany, in the years 2018 to 2020. Among those, both in-house patients and out-house patients were free to appear. Since the service is available every day and covered by governmental and private insurances, it is also open for self-payers or patients in social care. Approximately 30–40 dentists were deployed.

The emergency service times ranged from 17:00–22:00 on Mondays to Thursdays; Fridays from 13:00–22:00, Saturdays, Sundays and legal holidays from 8:00–22:00.

Retrospective data of the patients were collected, providing information about demographic details (sex, age, date of visit) as well as the diagnosis and therapy. All those data were extracted from the hospital ‘s dental documentation software (SAP, Walldorf, Germany; Visident, Wolfsburg, Germany) and underwent further analysis.

Patients who were treated or hospitalized for oral and maxillofacial surgical problems were excluded from this study since not every emergency dental facility has an affiliated OMFS department and we aimed to ease comparability.

The hospital law (Landeskrankenhausgesetz §§36, 37) of the State of Rhineland-Palatinate, Germany, explicitly allows the evaluation of existing patients data for retrospective study purposes. The study was approved by the local Ethics Committee (Ethik-Kommission Rheinland-Pfalz; 2020–15323-retrospektiv). Every patient signed an informed consent form about anonymized record use for retrospective research at the time of seeking treatment in the hospital. All methods were carried out in accordance with relevant guidelines and regulations. Microsoft office excel (Redmond, WA, USA) was used for data extraction and collection.

In our study, emergencies were subdivided into absolute and relative emergencies. To our knowledge, there is no internationally authoritative classification of dental emergencies according to severity. For this reason, the authors chose a comparatively simple dichotomous classification of the German Society of Dentistry and Oral Medicine (DGZMK). Here, "accidental injuries in the dental, oral and maxillofacial region, secondary bleeding after dental surgery and febrile, purulent inflammations originating in the dental system" are included in “absolute emergencies” In contrast, the other category consists in “relative emergencies”, which do not require urgent treatment [4].

Trauma was investigated separately from absolute emergencies during the waves. Included were emergencies like dentoalveolar trauma, soft tissue injury and (suspected) jaw fractures.

In addition to the inter-year comparisons, the first two waves of outbreak were examined in detail. Here, March 1st – June 15th 2020 were selected by a federal government agency and research institute for disease control and prevention as the period of the first wave (106 days) and October 1st as the start of the second wave continuing until the end of year 2020 (91 days).

Under-utilization was defined as the ratio of visits in 2020 to the mean number of visits in 2018 / 2019.

### Statistics

Further, moving averages of the daily number of visits were calculated, separately for patients with or without absolute emergency. The sliding window was set to three weeks to lessen the effect of weekly trends. The moving averages of 2020 were then compared to the mean of the years 2018 and 2019. For the construction of pointwise confidence intervals, we assumed that the number of visits for each particular time frame of three weeks would be distributed approximately evenly across the three years—if not for COVID-19. Analyses were performed in R 4.0.3 (R Foundation for Statistical Computing, Vienna, Austria. https://www.R-project.org/).

To calculate odds ratios (OR) – i.e. the change in odds of absolute and relative emergencies between 2020 versus the combined years 2018 and 2019 – univariate logistic regressions were performed. The depending variable was the type of emergency, the predictor was an indicator variable (0 = “visit in 2018 or 2019” and 1 = “visit in 2020”). The same method was also applied to subsets of the data set; stratified by sex, age group or month of the year. Further investigated subsets were defined by first and second wave compared to their respective reference timeframes in previous years.

Changes in absolute numbers were investigated with binomial tests, with the null hypothesis that a third of the total occurred in 2020. This was applied to the total number of patients, absolute emergencies, relative emergencies and trauma patients separately.

Due to the explorative character of this study, no adjustment for multiple testing was applied. All hypothesis tests were performed with a two-sided local significance level of 5%.

## Results

Our study includes a total of 11,219 dental emergency patients over a period of three years (Table 1).

**Table 1:** Comparison of visits per year

	Absolute	Relative	Total	Proportion absolute	OR; 2020 vs 2018&2019
2018	1137	3077	4214	26.98%	
2019	1213	2884	4097	29.61%	
2020	927	1981	2908	31.88%	1.19 (*p* < 0.0001)

In the years 2018 and 2019 that were not affected by COVID-19 in Germany, 4,214 (2018) and 4,097 (2019) patients presented at the emergency service. In 2020, 2,908 patients visited the emergency service (Table 1). Thus, the total count of cases decreased by more than 25%. An inverse relationship between the 7-day-incidence and the emergency visits was observed.

The share of absolute emergencies in 2020 was higher than in the years before (OR = 1.19, *p* < 0.0001; Table 1).

This effect was pronounced especially during times of higher incidence rates of COVID-19.

Looking at the distribution month by month, there is a strong contrast to the previous years in March 2020 (OR = 1.62, *p* = 0.002) and in December 2020 (OR = 1.59, *p* = 0.003) (Table 2).

**Table 2:** Comparison of visits per month

Month	Absolute Mean 2018/19	Absolute 2020	Total Mean 2018/19	Total 2020	Proportion absolute Mean 2018/19	Proportion absolute 2020	*p*-value	Odds Ratio
1	84.5	67	324.5	271	26.04%	24.72%	0.677	0.933 (0.673;1.294)
2	81.5	87	303.0	293	26.9%	29.69%	0.381	1.148 (0.843;1.562)
3	112.5	88	394.5	224	28.52%	39.29%	0.002	1.622 (1.19;2.21)
4	117.0	73	391.0	216	29.92%	33.8%	0.275	1.196 (0.867;1.648)
5	112.5	84	389.5	263	28.88%	31.94%	0.348	1.155 (0.854;1.563)
6	97.5	70	346.5	200	28.14%	35%	0.062	1.375 (0.984;1.921)
7	94.0	80	285.0	261	32.98%	30.65%	0.505	0.898 (0.655;1.232)
8	95.5	85	320.0	248	29.84%	34.27%	0.201	1.226 (0.897;1.675)
9	104.5	64	336.0	208	31.1%	30.77%	0.928	0.985 (0.703;1.379)
10	94.5	84	306.5	250	30.83%	33.6%	0.428	1.135 (0.83;1.553)
11	89.0	64	308.5	195	28.85%	32.82%	0.291	1.205 (0.853;1.703)
12	92.0	81	450.5	279	20.42%	29.03%	0.003	1.594 (1.175;2.163)

Men attended the emergency service marginally more often, with a male to female ratio of 1.16:1 over all three years (Tables 3 and 4).

**Table 3:** Sex comparison

Year	Sex	Absolute	Relative	Total	Proportion absolute
2018	F	493	1,479	1,972	25.00%
2018	M	644	1,598	2,242	28.72%
2019	F	494	1,385	1,879	26.29%
2019	M	719	1,499	2,218	32.42%
2020	F	414	942	1,356	30.53%
2020	M	513	1,039	1,552	33.05%

**Table 4:** Sex comparison – odds ratio

Sex	*p*-value	Odds Ratio
F	0.000	1.275 (1.113;1.462)
M	0.068	1.122 (0.992;1.269)

There was almost no difference between the average age in 2018, 2019 and 2020 (36.64–36.88 years). The number of patients visiting the emergency service was highest in age group 21–40 years (Tables 5 and 6).

**Table 5:** Age comparison

Age Group	Absolute Mean 2018/19	Absolute 2020	Total Mean 2018/19	Total 2020	Proportion absolute Mean 2018/19	Proportion absolute 2020
[0,20]	479.5	373	902.5	652	53.13%	57.21%
(20,40]	332.0	253	1,552.5	1,050	21.38%	24.1%
(40,60]	218.0	165	1,136.0	805	19.19%	20.5%
(60,120]	145.5	136	564.5	401	25.78%	33.92%

**Table 6:** Age comparison – odds ratio

Age	*p*-value	Odds Ratio
[0,20]	0.073	1.179 (0.985;1.413)
(20,40]	0.067	1.167 (0.989;1.377)
(40,60]	0.422	1.086 (0.888;1.327)
(60,120]	0.002	1.478 (1.156;1.89)

### Distinction of emergencies by severity

**Fig. 1:**
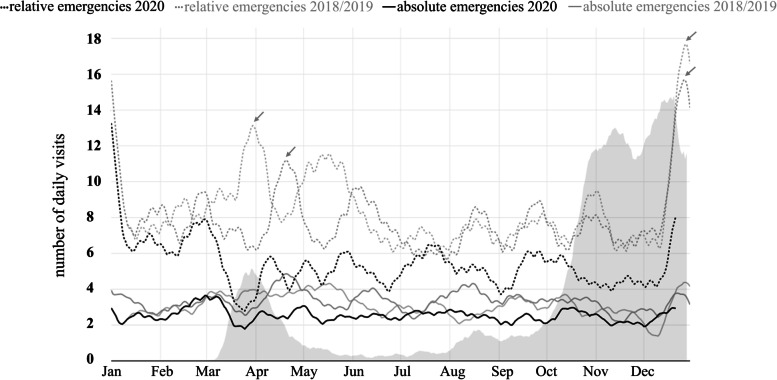
Smoothed rolling average of the number of daily visits that contained an absolute emergency (solid), and a relative emergency diagnosis (dashed) in the years of 2020 (black) and 2018/2019 (grey). Seven-day-incidence of COVID-19 in Rhineland-Palatinate in 2020 (grey shaded area). The Easter and Christmas Holidays are marked with “ → ” in the relative emergency line in 2018 and 2019

Both relative and absolute emergencies decreased during phases of high COVID-19 incidence. This effect was more distinctive for the relative emergencies. Figure 1 shows a clear decrease for absolute and relative emergencies simultaneously to the first peak of COVID-19 around the end of March 2020. This effect of decrease is missing for the absolute emergencies at the second peak at the end of the year. Here, only a decline in relative emergencies occurred. However, this was distinctly less pronounced than in spring. The chart shows a slow rise of relative emergencies between May and August with visits always staying below the level from 2018/19. During summer, absolute emergencies in 2020 have also almost completely normalized compared to the previous year's periods.

In 2020, the ratio of absolute emergencies to relative emergencies was significantly higher in women (*p* = 0.006) than in men (*p* = 0.068) (Tables 7 and 8).

**Table 7:** Comparison of first wave periods

Year	Absolute	Relative	Total	Proportion absolute	OR; 2020 vs 2018&2019
2018	404	1,072	1,476	27.37%	
2019	388	881	1,269	30.58%	
2020	281	528	809	34.73%	1.312 (*p* < 0.001)

**Table 8:** Comparison of second wave periods

Year	Absolute	Relative	Total	Proportion absolute	OR; 2020 vs 2018&2019
2018	264	801	1,065	24.79%	
2019	286	775	1,061	26.96%	
2020	229	495	724	31.63%	1.326 (*p* = 0.003)

The peaks of relative emergencies around Easter and Christmas holidays were markedly less prominent than in the years before (Fig. 1).

In patients 0–20 years old, the ratio of absolute versus relative emergencies was comparatively high in all years and COVID-19 independent (Table 5).

**Fig. 2:**
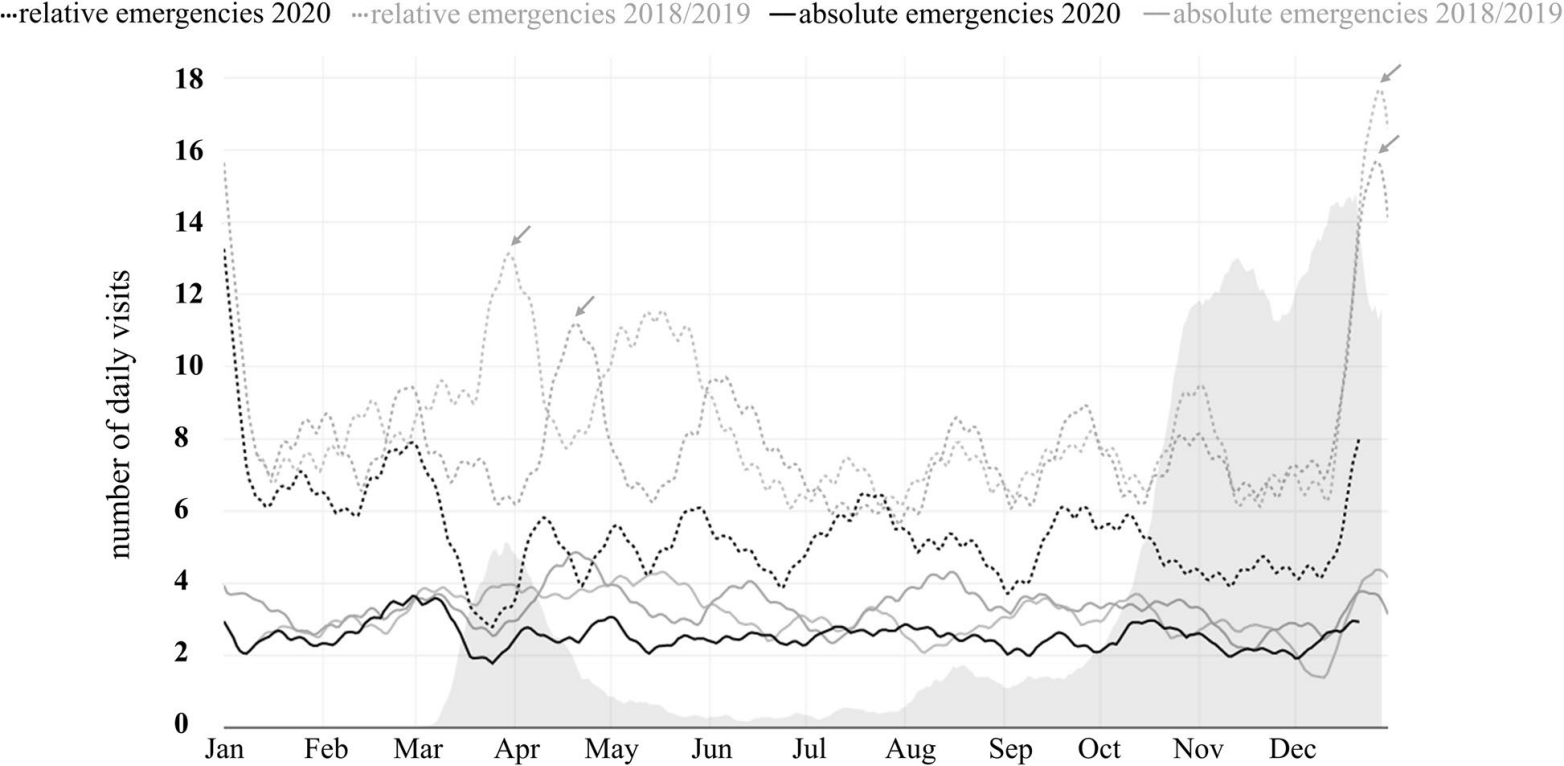
Smoothed rolling average of the number of daily visits of patients up to 60 years of age that contained an absolute emergency (solid), and a relative emergency diagnosis (dashed) in the years of 2020 (black) and 2018/2019 (grey)

Regarding the 0–60 years old group, the distribution of absolute and relative emergencies did not significantly differ between the 0–60 years old group and the entire study population (Fig. 2).

**Fig. 3:**
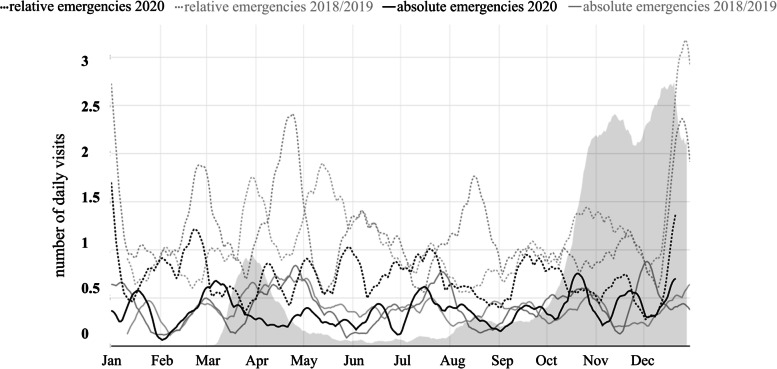
Smoothed rolling average of the number of daily visits of patients older than 60 that contained an absolute emergency (solid), and a relative emergency diagnosis (dashed) in the years of 2020 (black) and 2018/2019 (grey)

However, we found a different pattern for the age group 61 years and above (Fig. 3). Here, the number of absolute emergencies is almost equal to the non-COVID-19 years, whereas the total number of elderly visitors (over 60 years) decreased by 28.96%. Due to the large extent of under-utilization, the share of absolute emergencies rose significantly in this age group (*p* = 0.002) (Table 6).

### Under-utilization

**Fig. 4:**
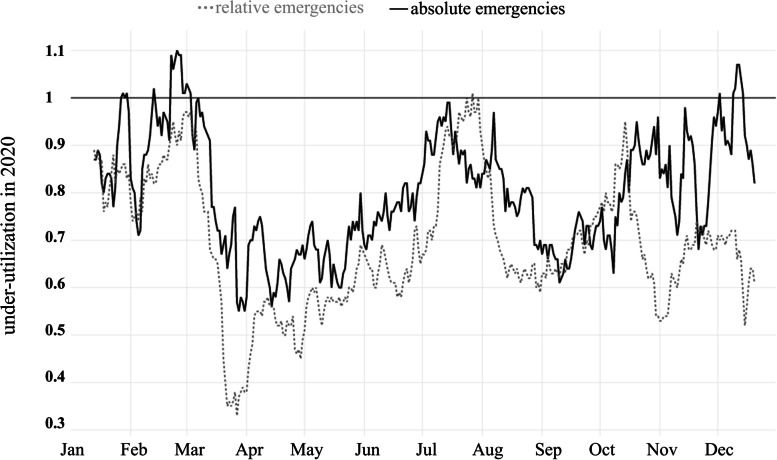
Ratio of visits in 2020 to the mean number of visits in 2018 / 2019. Visits with absolute emergencies in black, visits with relative emergencies in grey

Figure 4 shows the under-utilization in 2020 compared to the previous years. The first appearance of under-utilization was found in the beginning of March 2020, regarding both absolute and relative emergencies. This corresponds with the start of the pandemic situation in Germany and a lot of other European countries. From this point on, especially the relative emergencies show a more drastic drop than the absolute emergencies. During European summer (July and August), the observed effect of under-utilization fades again. Regarding the second wave, the effect of under-utilization was distinctly less pronounced compared to the first wave, considering absolute emergencies. On the contrary, an even larger under-utilization effect for relative emergencies is detectable (Fig. 4).

This resulted in an odds ratio for an under-utilization of 1.312 (1.111; 1.550, *p* < 0.001) during the first wave of outbreak and an odds ratio for an under-utilization of 1.326 (1.087; 1.594, *p* = 0.003) during the second wave of outbreak (Tables 7 and 8).

### Inter-wave comparisons

**Fig. 5:**
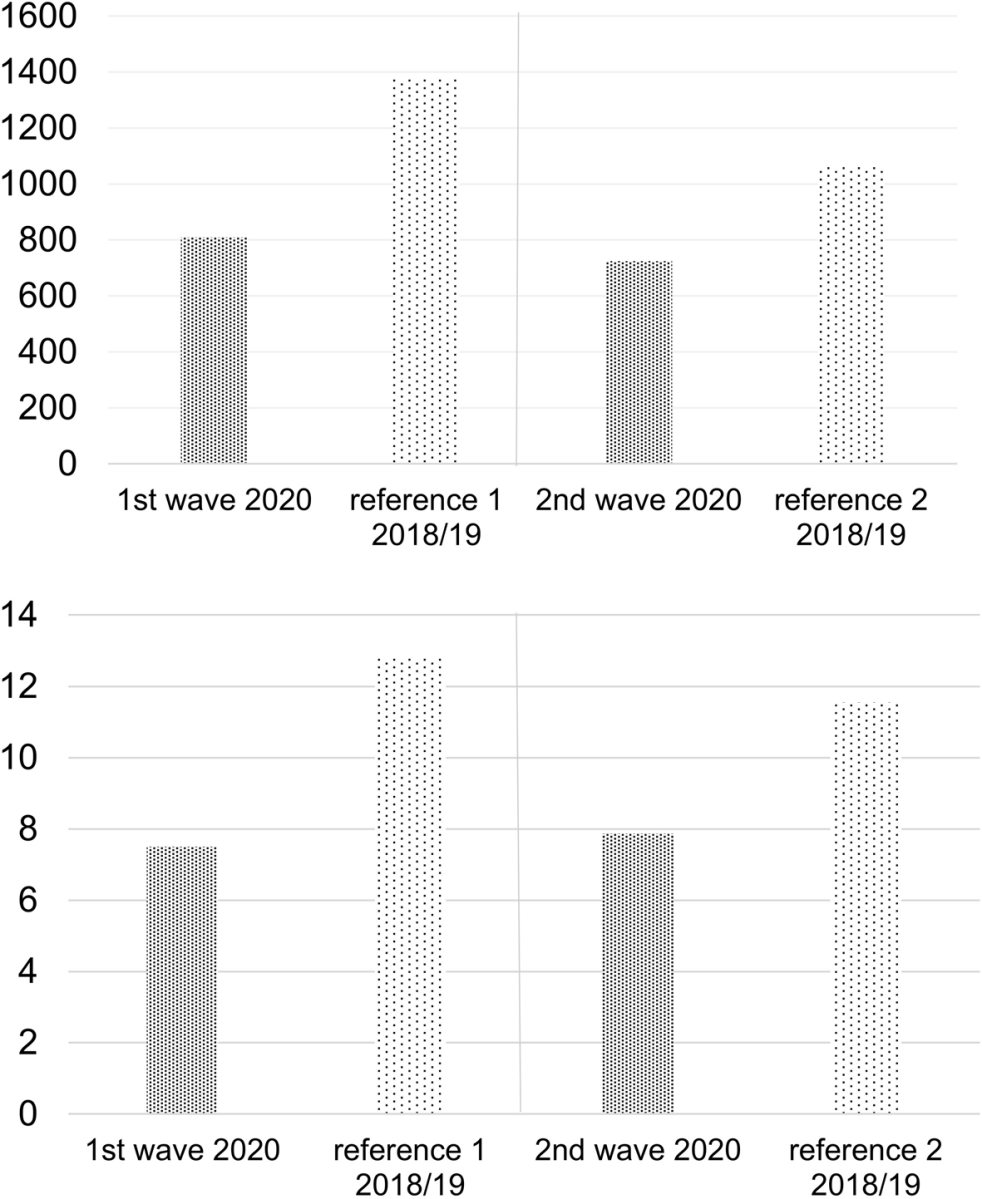
**a** Total number of patients during the waves of outbreak (1^st^ and 2^nd^ wave). **b** total number of patients during the waves of outbreak per day. The figure shows the total number of patients during the time period of the first and the second wave of COVID-19 in 2020 and the average number of the exact same time period of/in 2018 and 2019 as a reference

During first and second wave, fewer patients visited the emergency service (*p* < 0.0001 for both waves; Fig. 5; Tables 7 and 8). The difference was larger between the first wave and its corresponding time period compared to the second wave (Fig. 5; Tables 7 and 8). In addition, it should be mentioned that in the reference time of the first wave more people visited the dental emergency service than in the respective reference time for the second wave. To account for the slightly different duration of the waves, Fig. 5 shows the differences per day.

**Fig. 6:**
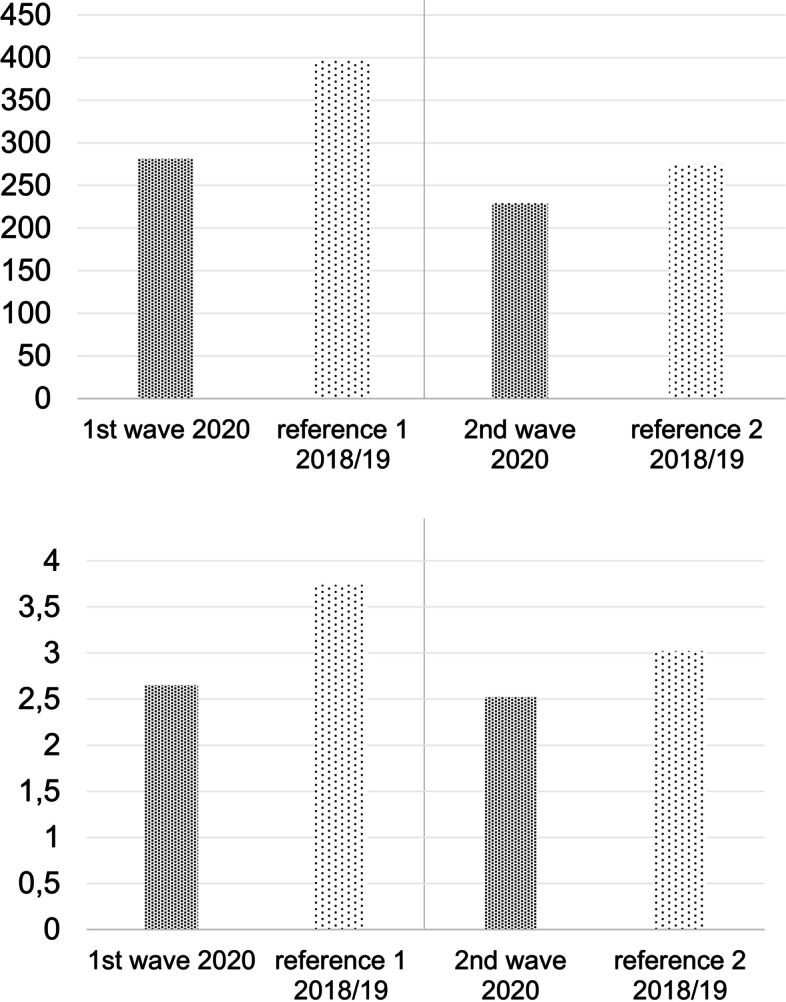
**a** Absolute emergencies during the waves of outbreak (1^st^ and 2^nd^ wave). **b** absolute emergencies during the waves of outbreak per day. The figure shows the number of absolute emergencies during the time period of the first and the second wave COVID-19 in 2020 and the average number of the exact same time period of 2018 and 2019 as a reference

The number of absolute emergencies was lower during the waves. The decrease both in total and per day was greater during the first wave as compared to the second wave (-29.04% (*p* < 0.0001) compared to -16.73% (*p* = 0.02)). There was still a significant reduction observed during the second wave, however, less pronounced than during the first wave. (Fig. 6; Tables 7 and 8).

**Fig. 7:**
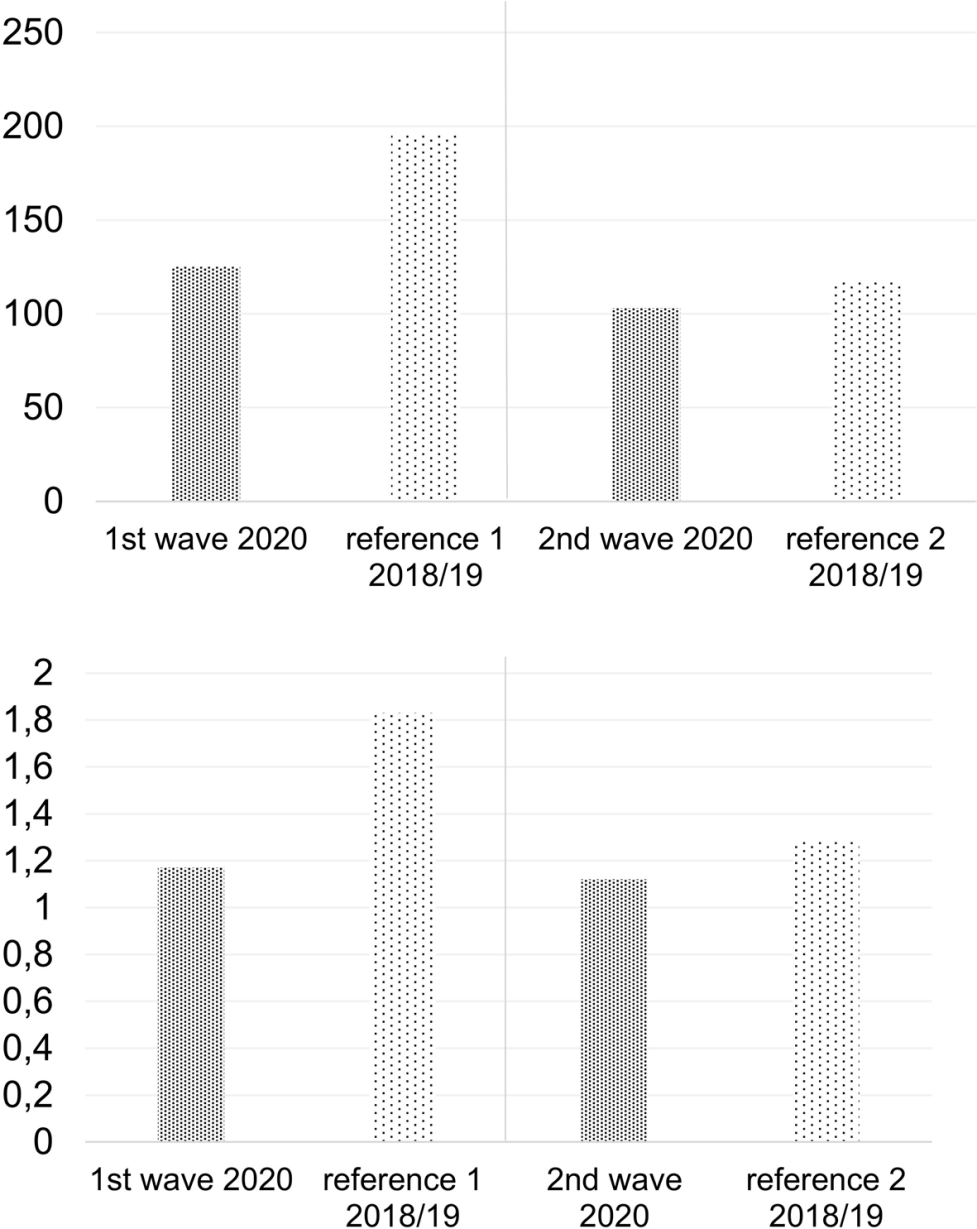
**a** Trauma during the waves of outbreak (1^st^ and 2^nd^ wave). **b** trauma during the waves of outbreak per day. The figure shows the number of trauma during the time period of the first and the second wave COVID-19 in 2020 and the average number of the exact same time period of 2018 and 2019 as a reference

The wave-dependent trauma occurrence was shown in Fig. 7.

Again, a significant difference between the first wave and its reference time period was found (-36.22% and *p* < 0.0001). During the second wave, the difference to the previous years was comparatively small and not significant (-12.34% and *p* = 0.07; Tables 7 and 8).

## Discussion

Our study revealed an interplay of pandemic and seasonal parameters and number of visits: relative and absolute emergencies decreased during COVID-19. This effect was considerably more pronounced regarding the relative emergencies, while the number of patients with absolute emergencies remains rather constant, even in times of pandemic-related contact restrictions. Variations were also found in different age groups. The share of absolute emergencies, for example, increased significantly in the elderly age group (61–120 years old) in phases of high COVID-19 incidence.

Therefore, our study provides an overview of the situation in dental emergency service before and during the COVID-19 pandemic in a dental unit of a university hospital. The comparison of the pandemic waves with each other and with previous years' periods are clear strengths and special features of our study.

Our data show that the total count of cases decreased by more than 25% in 2020. We found an approximately inverse dependency of 7-day-incidence of COVID-19 and number of visits (Fig. 1). This effect has also been found in other studies regarding dental and non-dental emergencies during the pandemic [5–7]. Other investigators found the absence of patients in the pandemic being triggered by the fear of infection [8, 9]. Therefore, this fear might have contributed to the decrease in emergency visits during the pandemic in our study.

The inverse dependency of the COVID-19 incidence and the number of visits was not observed in all studies. In another investigation, 724 patients were treated in a dental emergency service of a university hospital in April 2020, whereas only 160 patients received dental care in 2019 during the same time period and at the same affiliation [10]. It can be speculated that the under- and over-utilizations, respectively, strongly depend on the political measures taken in the health sector of the respective region. A study that compared three periods (pre-lockdown, lockdown, post-lockdown) in a university hospital demonstrated that the daily number of patients increased during the lockdown [11].

In our study, emergencies were subdivided into absolute and relative emergencies as suggested by a large European dental society. This dichotomous classification exists since 1994 and is simple and unambiguous. It clearly assigns diagnoses to grades of urgency.

Unfortunately, there is no widely agreed classification system for dental emergencies with regard to urgency of the required treatment.

In the study by Famà et al. patients were subdivided according to the Glasgow Coma Scale. The patients were divided into three groups according to the urgency of treatment [12, 13]. Another study also used a three-level classification system. However, this study included maxillofacial patients, whereas our study focused exclusively on dental outpatients [14, 15]. In previous studies on this topic, cases were often divided into urgent and non-urgent or not further classified [7, 11].

In the study by Famà et al., as for the group of most urgency, the number of visits did not differ between pre-pandemic and pandemic phases [12]. This is consistent with our finding that the number of absolute emergencies was less affected during 2020. The least-urgent cases of this study decreased like the relative emergencies in our study [12]. In the study by Guo et al. a decrease of relative emergencies by more than 50% was noted, which again supports our observation [7]. In that study, a similar volume of patients (12,416) were included, as in our investigation. However, our study did not only compare periods of low and high incidence during the pandemic as occurred in the study by Guo et al., but also analyzed waves of the COVID-19 pandemic and corresponding periods of previous years. Despite the use of different classification systems, both Guo et al. and our study found an inverse dependency between the number of visits and COVID-19 incidence [7].

As shown, the share of relative emergencies in a similar setting is about 75% [16]. This matches our findings of approximately 72% for the non-COVID-19 years (Table 1). In 2020, proportionally more absolute dental emergencies than in 2018 and 2019 were documented, while the relative emergencies decreased compared to previous years.

Nevertheless, it should be taken into account that the political measures were different among countries and regions. On some continents, for example, curfews lasted for weeks, whereas in several European countries, curfews were limited to the nighttime and shorter periods.

Like our workgroup, Lentge et al. compared the COVID-19 situation with the two previous years (2020 with 2018/19) in a cohort under similar political conditions. In contrast, the observation period lasted four weeks only. Here, craniomaxillofacial surgery patients were included only and the number of patients in 2020 was significantly lower compared to both years before [5].

In our study, the proportion of absolute emergencies was highest in the age group 0–20 years, i.e. twice as high as compared to some other age groups (Tables 5 and 6), which might have been caused by the increased likelihood of traumatic events in in childhood and youth [17–19].

Patients older than 60 years who only suffered from relative emergencies rather tended to avoid visiting the emergency service during 2020 (Fig. 3, Tables 5 and 6). This concurs with the results by Howley et al., who have shown a reduction of emergency visits by elderly people during COVID-19. Overall, a decrease in emergency visits by 16% was observed in patients of 70 years and older [20]. In our study, unlike the relative emergencies, the absolute emergencies decreased to a lesser extent in the elderly age group. In the younger patients and in the total cohort, there was a rather similar reduction in absolute and relative emergencies (Figs. 1, 2, 3). Possibly, fear of infection in an emergency office may vary among age groups.

In non-COVID-19-years and during the pandemic, slightly more men presented the dental emergency service, which is in agreement with the findings by other investigators [14, 21]. The pandemic showed no significant effect on gender distribution.

Our results show a clear overall increase of patient numbers during the Easter and Christmas holidays (Fig. 1). This concurs with the findings of another study that investigated emergency services during Christmas holidays under non-pandemic circumstances [22]. This result could be linked to the fact that many private dental offices were closed for vacation during the periods mentioned. In addition, the rise of emergency visits could have been caused by an increase in leisure time activities and thus a higher risk for injuries [18, 23].

The reduction of total emergency visits in our study by 41.06% during the first wave and by 31.86% during the second wave (Fig. 5) is consistent with the study by Cordova et al. who reported a decrease in a plastic surgery emergency services during the pandemic waves [24]. Similarly, in an ophthalmologic emergency service, a reduction of 53% was noted [25]. However, although both studies also compared COVID-19 versus non-COVID-19 phases, they did not distinguish between different COVID-19 waves of the pandemic. In the second wave, absolute emergencies were less reduced than in the first wave as our study revealed.

The number of patients presenting after traumatic events decreased by approximately two thirds less in the second wave compared to the first wave. Possibly, people were more likely to avoid outdoor activities and sports during the first wave, whereas such activities might have played a greater role again during the second wave. That recreational activities are associated with dental trauma has been reported before [18, 23].

Like most studies on this topic, our investigation had a retrospective character. Moreover, like most, if not all, studies on this topic, our study describes the situation at a single center. Clear strengths of our study are the large number of patients (*n* = 11,219) and the use of more than one non-COVID-19 year as a reference for the pandemic year. Furthermore, a distinction was made in our study between absolute and relative emergencies on basis of the recommendations of a large scientific society. These features of our study make it one of the most comprehensive investigations on outpatient dental emergency services during COVID-19 in industrialized countries and helps predict patient behavior and needs in a future pandemic situation.

## Conclusions

In a pandemic situation, dental emergency facilities can expect fewer patients overall, but relatively more absolute emergencies. It can be assumed that there is an acclimatization effect regarding the wave comparison, as visits increased less in the second wave compared to the first wave. Patients older than 60 years who only suffered from relative emergencies rather tended to avoid visiting the emergency service during 2020.

Since the COVID-19 pandemic is still ongoing and other pandemic situations could follow, this study helps implement public health strategies by prediction of patient behavior regarding dental emergencies.

## Data Availability

The datasets generated and analyzed during the current study are not publicly available because conclusions could possibly be drawn about individual behavior. However, data are available from the corresponding author on reasonable request.

## Abbreviations

WHO: World Health Organization
DGZMK: German Society of Dentistry and Oral Medicine
OR: Odds ratio
